# Risk Factors for Infectious Diseases in Urban Environments of Sub-Saharan Africa: A Systematic Review and Critical Appraisal of Evidence

**DOI:** 10.3390/tropicalmed4040123

**Published:** 2019-09-29

**Authors:** Matthew R. Boyce, Rebecca Katz, Claire J. Standley

**Affiliations:** Center for Global Health Science & Security, Georgetown University, Washington, DC 20057, USA; Matt.Boyce@georgetown.edu (M.R.B.); Rebecca.Katz@georgetown.edu (R.K.)

**Keywords:** Sub-Saharan Africa, urbanization, infectious disease, communicable disease, risk factors, systematic review

## Abstract

Our world is rapidly urbanizing. According to the United Nations, between 1990 and 2015, the percent of the world’s population living in urban areas grew from 43% to 54%. Estimates suggest that this trend will continue and that over 68% of the world’s population will call cities home by 2050, with the majority of urbanization occurring in African countries. This urbanization is already having a profound effect on global health and could significantly impact the epidemiology of infectious diseases. A better understanding of infectious disease risk factors specific to urban settings is needed to plan for and mitigate against future urban outbreaks. We conducted a systematic literature review of the Web of Science and PubMed databases to assess the risk factors for infectious diseases in the urban environments of sub-Saharan Africa. A search combining keywords associated with cities, migration, African countries, infectious disease, and risk were used to identify relevant studies. Original research and meta-analyses published between 2004 and 2019 investigating geographical and behavioral risk factors, changing disease distributions, or control programs were included in the study. The search yielded 3610 papers, and 106 met the criteria for inclusion in the analysis. Papers were categorized according to risk factors, geographic area, and study type. The papers covered 31 countries in sub-Saharan Africa with East Africa being the most represented sub-region. Malaria and HIV were the most frequent disease focuses of the studies. The results of this work can inform public health policy as it relates to capacity building and health systems strengthening in rapidly urbanizing areas, as well as highlight knowledge gaps that warrant additional research.

## 1. Introduction

Catalyzed by the Industrial Revolution in the 19th century, urbanization continues to be a major driver of demographic change in today’s world. By 2010, for the first time in history, the majority of humans resided in cities [1]. Estimates suggest that the trend of increasing urbanization will continue, and that over 68% of the world’s population will call cities home by 2050 [2]. Although currently the least urbanized continent, by some measures Africa, and particularly sub-Saharan Africa, is the most rapidly urbanizing region of the world, with estimates suggesting an increase in the proportion of the population living in urban areas from 40% to between 56–62% of the total between 2010 and 2050 [2]; only Asia comes close to matching this projected rate [1,3]. However, this reality is complex, and urbanization in sub-Saharan Africa is distinct from current or historical trends of urbanization in other parts of the world [4]. 

Concurrent with these demographic changes, sub-Saharan Africa continues to experience changing epidemiological patterns, particularly a shift towards a “dual” burden of disease [5]. Non-communicable diseases (NCDs) are becoming more prevalent even while infectious diseases remain a substantial source of morbidity and mortality, especially among children; moreover, many NCDs, including diabetes, obesity, and heart disease, are strongly associated with urbanization [6,7]. The relationship between urbanization and infectious disease is less clear-cut and is moreover impacted by external factors such as geography and climate. In some cases, urbanization may directly contribute to the emergence or re-emergence of infectious diseases through the degradation of ecosystems, intensification of agriculture, and increased opportunities for the human-animal interface, especially with rodent or peri-domestic reservoir species [8]. Higher population density, poor housing, and poor sanitation infrastructure—as compared to rural areas—have also been shown to increase the risks associated with some infectious diseases [3]. Conversely, the urban environment may reduce the transmission and incidence of other pathogens, for example, through reducing habitats for some vectors [8]. Moreover, urban environments are not homogeneous, so risk factors are likely to vary across the continent, across a country, and even within a city [9,10].

Aside from drivers of disease emergence, urban environments present new challenges for the management of infectious diseases. Provision of new or supplementary control measures may not be able to follow the same model used for expanding health services to rural settlements, and scaling up to meet larger population demands may strain existing systems. Informal settlements (or “slums”), which are strongly associated with rapid urbanization, are commonly overcrowded and lack basic services and facilities, factors which the WHO attributes to a higher risk of certain communicable diseases, notably diarrheal illness [11]. However, residents of informal settlements may (but not always) have better access to healthcare than rural communities, potentially offsetting some of the disease risk [12]. Moreover, cities themselves are highly heterogeneous, with urbanization also associated with increasing inequality among citizens [13]. Therefore, the widespread practice of using average values for urban population indicators, including health, may hide important differences in risk factors and access to health care, and consequently control opportunities between disparate groups of urbanites [12].

To explore these issues further, we sought to conduct a comprehensive review of urbanization in sub-Saharan Africa and its impact on infectious diseases. Our objectives were to highlight geographic and behavioral risk factors for infectious diseases in urban settings studies, describe interventions that have specifically been applied to cities, and identify current gaps in the literature which may constitute opportunities for future research in this area. 

## 2. Methods 

### 2.1. Literature Search

We conducted a systematic search in the PubMed and Web of Science databases on risk factors for infectious diseases in urban environments in sub-Saharan Africa. Synonyms for “city, “travel,” “communicable disease,” and “risk” were combined with a list of priority disease types and countries in sub-Saharan Africa to identify relevant studies. The references of these studies were reviewed to identify additional studies worthy of inclusion. A supplementary file provides the complete search syntax (Supplementary File 1). Our last search was conducted in July 2019. In an effort to limit bias, we did not place any restrictions on the language of publication. 

### 2.2. Study Selection

We determined that the articles included from the search should meet the following criteria: (i) discussion of infectious burden/transmission/control in urban settings; (ii) study location (at least one, for multi-country studies) in sub-Saharan Africa; (iii) published in or after 2004; and (iv) discuss geographic or behavioral risk factors, changing disease distributions, or control programs. Two authors collaborated on screening articles. One author screened titles and a second screening was performed by two authors on article abstracts. Discrepant results were discussed between the authors until a unanimous decision regarding inclusion was achieved. 

### 2.3. Data Analysis

Following our study selection, we characterized included studies based on their geographic location, study type, and infectious disease type. Studies were reviewed for geographic risk factors, behavioral risk factors, changing disease distributions, or discussion of interventions and control programs. For this study, we characterized diseases as enteric (e.g., cholera, hepatitis, polio), HIV, malaria, respiratory (e.g., measles, pneumonia, tuberculosis), viral hemorrhagic fever (VHF) (e.g., Ebola, flaviviruses, Lassa fever), or other (e.g., helminth infections, neglected tropical diseases, sexually transmitted infections other than HIV). Meta-analysis was impractical because of the diversity of study types and data, so we present the results of our search in the following narrative.

## 3. Results

**Figure 1:**
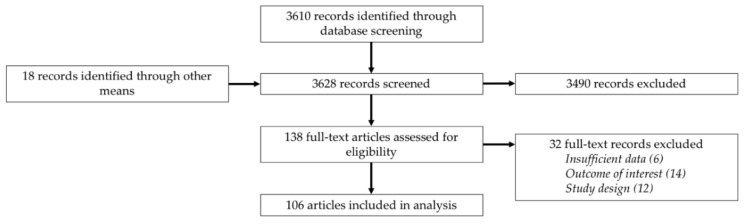
Preferred Reporting Items for Systematic Reviews and Meta-Analyses (PRISMA) study selection diagram.

A total of 3610 titles published from 2004 to 2019 were identified from the database searches. An additional 18 studies were identified through other means, leading to a total of 3628 studies. After screening these items and removing duplicates, a total of 138 articles that were assessed for eligibility. Of these, an additional 32 were excluded resulting in a total of 106 studies included in our analysis (Figure 1).

### 3.1. Study Characteristics

**Figure 2:**
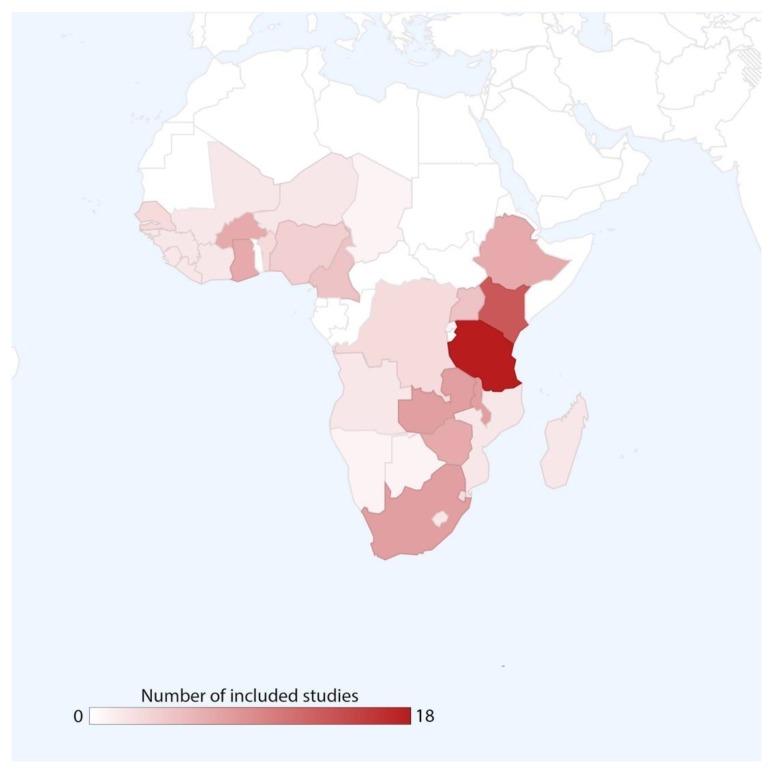
Geographic distribution of included studies (excluding review articles).

We identified studies covering urban environments in 31 sub-Saharan African countries, plus four review articles with a focus on sub-Saharan Africa as a whole. Two studies were conducted in Angola [14,15], three in Benin [16,17,18], one in Botswana [19], seven in Burkina Faso [20,21,22,23,24,25,26], five in Cameroon [16,25,27,28,29], one in Chad [30], two in Cote d’Ivoire [25,31], three in the Democratic Republic of Congo [15,25,32], three in Eswatini [25,33,34], seven in Ethiopia [25,35,36,37,38,39,40], seven in Ghana [25,41,42,43,44,45,46], two in Guinea [25,47], two in Guinea-Bissau [48,49], fourteen in Kenya [12,16,25,50,51,52,53,54,55,56,57,58,59,60], two in Lesotho [25,33], two in Liberia [25,47], two in Madagascar [61,62], seven in Malawi [25,33,63,64,65,66,67], one in Mali [25], two in Mozambique [68,69], one in Namibia [70], two in Niger [25,71], four in Nigeria [72,73,74,75], one in Rwanda [25], three in Senegal [25,50,76], two in Sierra Leone [25,47], eight in South Africa [77,78,79,80,81,82,83,84], eighteen in Tanzania [25,60,85,86,87,88,89,90,91,92,93,94,95,96,97,98,99,100,101], five in Uganda [60,102,103,104,105], eight in Zambia [16,25,106,107,108,109,110,111], and seven in Zimbabwe [25,33,56,86,112,113,114] (Table 1; Figure 2).

Of the included studies, 13 focused on enteric diseases, 34 focused on HIV, 38 focused on malaria, 12 focused on respiratory diseases, five focused on VHF, and 16 focused on other diseases (Table 1). Eight studies focused on multiple disease classifications. For more information on study details, see Supplementary File 2.

### 3.2. Urban Risk Factors for Infectious Diseases

Risk factors were categorized as geographic or behavioral. Several studies also discussed individual-level demographic risk factors (e.g., age, sex, etc.), but these were not considered further as they were beyond the scope of the review. We also assessed whether articles described changing epidemiology or control programs and other interventions in the context of sub-Saharan African urban infectious diseases. Table 2 provides a summary of the risk factors identified across each disease group considered, with further details provided in the following sub-sections. 

#### 3.2.1. Geographic Risk Factors

##### Population Density 

High population density was a frequently identified risk factor for infectious disease in sub-Saharan Africa. Diseases showing increased prevalence or transmission in high population density urban environments include respiratory diseases [48,49,71,115], viral hemorrhagic fevers [15,47,74], malaria [42,98,102], and enteric diseases [30,42,46]. Correspondingly, this places populations residing in specific high-density neighborhoods, and especially in informal settlements or slums, at an increased risk for infectious disease. This association holds even at the household level, with one study showing that larger households (i.e., those with more people) were at a higher risk for enteric disease [30]. 

##### The Built Environment

Characteristics of the built environment can also be important risk factors for disease. Evidence exists suggesting that malaria risk in urban areas is higher in irregularly or sparsely built-up areas [20,97], and that high building density reduces dengue risk [21]. Poor quality housing was found to be a risk factor for certain respiratory diseases [49], malaria [17,37,67,99,101], and soil-transmitted helminths [53]. More specifically, for malaria, there is evidence suggesting that the risk of malaria infection is lower among occupants of completed houses [67], brick houses (compared to mud houses) [17], houses with window screening or netting (compared to those with absent, or incomplete screening) [44,99], houses with electricity, and houses with piped water [67]. Having finished household floor material was also protective against soil-transmitted helminth infections [53], as was having multiple windows for tuberculosis [38].

##### Municipal Services

Related to population density, the lack of municipal services, such as hygiene and sanitation, and health services, are other major identified risk factors. Sasaki and colleagues found that insufficient drainage and lack of access to a latrine increased the risk for cholera in Lusaka, Zambia [110,111]. Keating and colleagues found similar results for malaria whereby households in well-drained areas had a significantly lower risk of infection [57]. Furthermore, while the risk of malaria infection is generally considered to be lower in urban versus rural environments [27,31,98,101,105,116], within urban settings the risk for malaria was shown to be impacted by low quality water supplies and sanitation facilities [42,76]. In addition to access to hygiene facilities, there is evidence that the quality of facilities also impacts the risk for enteric disease [42]. Other types of municipal services, such as waste disposal, can also have an impact; studies have demonstrated increased risk of dengue virus exposure in households closer to dumpsters [21]. Greater distances to health care facilities were also shown to be associated with higher burdens of malaria in urban environments [14], while closer distances [107] or proximity to major roads [63] were associated with reduced risk of HIV. Evidence also exists that women living in urban neighborhoods near a market may be at an increased risk of HIV infection [107].

##### Climate and Natural Environment

The climatic and natural environment of cities has been shown to impact both the prevalence of existing infectious diseases as well as the emergence of new ones. Several studies showed that the relative wetness and proximity to water in urban environments increased the risk for malaria [14,20,26,37,76,97,102,114,117,118,119] and certain VHFs (e.g., dengue) [36], as would also be the case in non-urban settings. Similarly, temperature was also identified as a risk factor in urban areas [37,98]. However, in urban areas, population density may act synergistically with these risk factors to increase transmission risk. Other work has also demonstrated that populations residing at lower elevations are at higher risk for enteric disease [54,113] and soil-transmitted helminth infections [53].

Urban communities that live in closer proximity to certain animal populations can also be at higher risk for malaria [117], viral hemorrhagic fevers [74], and other infections such as leptospirosis [87]; more generally, the nature and frequency of human-animal interactions in urban environments are different from rural settings and may prompt the “spillover” of pathogens, and result in the emergence of new human diseases. Other factors that are predicted to impact the risk of disease, and specifically the emergence of disease, include proximity to agriculture or dense vegetation [18,26,37,61,97] and greater wetness variability [18].

#### 3.2.2. Behavioral Risk Factors

In conjunction with geographical and physical risk factors, the risk of infectious disease in urban environments is impacted by a range of behavioral risk factors. 

##### Hygiene and Sanitation Practices

Related to the lack of availability of appropriate hygiene and sanitation services, personal hygiene behaviors, such as poor handwashing practices [30,110,111], drinking water without proper treatment (i.e., chlorination) [31,46,53,110], and outdoor play [54] were all shown to increase the risk for infection with enteric diseases, such as typhoid, as well as for helminth infections. Improper household waste management, likely in conjunction with municipal-level failings, can also increase the risk of vector-borne diseases such as dengue and malaria [21,76,118].

##### Sexual Practices and Behaviors

Although not unique to urban environments, sexual practices and behaviors were also shown to be an important risk factor for HIV and other sexually transmitted infections (STIs). Studies showed that riskier sexual behaviors increased the risk for HIV infection in urban settings [16,56], though there is evidence that even the poorest of urban populations residing in slums use condoms more frequently and have better access to testing for STIs when compared to more rural populations [12]. Work has also shown that populations residing in urban slums may be less likely to know about appropriate HIV prevention practices [12] than their non-slum or rural counterparts. Other sexual behavior risk factors include having more sexual partners [24,35,41,79,86,89,92,107], non-marital sexual partners [86,89,107], sexual debut before the age of 16 years [59,64,89,92], not using protection (i.e., wearing a condom) [28,35,77,78,83,112], consuming alcohol before sex [35,106,112], having transactional sex [69,79,112], having anal sex [28], having sex during menses [35], and being uncircumcised [69]. Evidence regarding the effects that marriage has on risk suggests that marriage or a history of marriage (i.e., divorce) increases the risk of disease [23,24,58,59,68], although some work has also demonstrated that it is associated with a decrease in risk [88]. Having a first sexual partner aged 24 years or older was also shown to increase the risk of HIV [24], as was a history of sexual abuse [35,77]. One study also found that the use of oral contraceptives increased the risk for herpes infection [23], likely due to subsequent effects on the use of condoms or other forms of protection.

##### Human Movement

Human movement was another significant risk factor and encompassed several different behaviors. The risk for multiple diseases is increased as a result of travel, including travel by urbanites to areas where certain disease prevalence (e.g., malaria) is higher [15,34,40,51,60,61,62,66,67,93,117], as well travel to cities by other populations [50,72]. Traveling and spending time away was also associated with an increase in HIV risk [29,92]—possibly as a result of this more mobile population reporting more risky sexual behaviors (i.e., more partners and one-off contacts) [29]. This association was also true if a sexual partner traveled [89] or if their partner’s profession is one traditionally involving travel (e.g., driver, truck driver, or soldier) [88]. One study also found that traveling for school or employment increased the odds for respiratory infection [103]. Proximity to migration routes was also identified as a risk factor for respiratory infections [71], and some evidence suggests that the risk for malaria in urban populations can be increased by driving, as tire tracks can leave artificial vector breeding sites [118]. Voeten and colleagues found that there was a strong, positive association between recent in-migration and HIV prevalence [120].

##### Education and Employment

Education and occupation were other identified risk factors associated with behavior. The risk of HIV was found to be higher among unemployed homeless populations [81], clerical/manual laborers (compared to professionals) [19], and among women who reported earning their own income [109]. One study conducted by Augusto and colleagues found that sex workers with other means of income had a lower risk for HIV infection [68]. The workplace of female sex workers was also shown to be a risk factor for hepatitis B infection [35]. Generally speaking, education was protective against infection with evidence suggesting that higher education is associated with lower risk for HIV [35,58,68,69,92], malaria [20,32,67,73,93], other STIs [100], and tuberculosis [38].

##### Socioeconomic Standing

Socioeconomic standing and wealth were also risk factors for urban disease. In urban environments in sub-Saharan Africa, wealthier populations are at a decreased risk for HIV [25,70,84], malaria [20,32,67,105], tuberculosis [38], and other diseases like Lymphatic filariasis [90]. Evidence regarding household size as a risk factor for disease in urban environments is incongruent. Msamanga and colleagues found that larger household size reduced the risk of HIV [88], but other work has shown that larger households are associated with increased risk for malaria [117] and respiratory diseases like tuberculosis [48,49].

Other behavioral risk factors for HIV in urban environments include religion [24], history of piercing with sharp materials and history of abortion [39], having dependents or a having steady partner [35], and being single [86]. Risk factors for hepatitis B include a previous history of blood transfusion, body tattooing, surgery and unsafe injection [39]. The risk for respiratory diseases was also impacted by a range of other behaviors including smoking [38], breastfeeding practices [48], and cooking behaviors that impact indoor air pollution [22].

#### 3.2.3. Epidemiological Changes and Changing Disease Burdens 

Of the included studies that considered shifting epidemiology or disease burdens in urban environments, most focused on malaria. Evidence suggests that residing in urban environments is generally associated with lower burdens of malaria [31,43,116,119], as well as other parasitic infections [31]. However, the lower prevalence in urban areas also causes reduced immunity over time, rendering urban populations more susceptible to infectious disease outbreaks upon exposure [118]. Studies also suggest that urban populations may be at heightened risk for viral hemorrhagic fevers [47] and that epidemiologic drifts are occurring—possibly due to consequences stemming from poor sanitation and overcrowding [74]. 

Evidence exists suggesting that, compared to rural areas, urban areas are at heightened risk for certain diseases including HIV [25,33,94] and tuberculosis [104]. Work also exists that specific urban populations, such as those living in slums, are at heightened risk for HIV when compared to other urban or rural populations [59].

The prevalence of one disease condition may impact the epidemiology of other infectious diseases. For example, the risk of HIV in some African cities has been associated with co-infection with herpes simplex virus [16]; likewise, the risk for respiratory infections, and specifically tuberculosis, can be impacted by the burden of HIV in urban environments [115] or by close contact with others infected with tuberculosis [38,104]. This contact may be the result of living with an ill individual, and indeed, living with an individual infected with a disease was also found to be a risk factor for dengue [36] and STIs [100]. Wong and colleagues also found that living with HIV-infected individuals was associated with increased rates of respiratory and diarrheal infections in HIV-negative individuals [52].

#### 3.2.4. Control Programs

Relatively few studies explicitly investigated control programs in urban environments. Urban populations frequently seek care at hospitals, clinics, chemists, and other drug vendors for infectious disease treatments, though a large proportion of visits were to unlicensed vendors [55]. This healthcare infrastructure can lower the risk of infectious disease outbreaks in urban populations, especially when services are provided free of charge [50], but reliance on unlicensed vendors or traditional healers can also limit opportunities for early outbreak detection or intervention. 

Several studies demonstrate the efficacy of insecticide-treated bed nets (INTs) in reducing the risk of malaria [17,20,26,32,34,73,96,99,101,102,114,118] and dengue [36] in urban settings. Indoor residual spraying also appears to be an effective means for preventing malaria in urban environments [76,114], as does environmental management [95], and larviciding [99], which has also been shown to remain cost-effective even as the incidence of malaria drops [85]. In one study, more febrile illness was reported in children from households using protective measures against mosquitoes [76]. Another study that examined the characteristics of malaria in Swaziland among travelers, found that chemoprophylaxis increased the risk of infection [34]. 

Access to treatment—or lack thereof—can also increase the risk of infection. One study conducted in Nairobi, Kenya found the reporting of deworming to be protective against soil-transmitted helminth infection [53]. Counterintuitively, another study showed that relative to no treatment, being on antiretroviral treatment for less than 6 months and 6–12 months was associated with an elevated risk of mortality in urban populations [82]. In rural populations, relative to no treatment, being on treatment for 6–12 months and greater than 12 months was protective. However, more evidence exists suggesting that not initiating treatment increases the risk for infection in urban populations. One study found that not initiating antiretroviral treatment (for women who required it) resulted in higher in-utero transmission rates of HIV [80]. Other studies showed that vaccination delays can be common in migrant populations found in urban environments, especially for recent (versus settled) migrants [72]. These delays in vaccination campaigns can result in a higher risk for a multitude of vaccine-preventable diseases [15,72], especially because evidence exists that vaccines reduce the risk of infection of their target diseases [38].

## 4. Discussion

The studies included in this review largely identify physical and human geographical risk factors which mirror those for other regions of the world. Many of these risk factors—such as hygiene and sanitation, housing conditions, human behaviors, and socioeconomic disparities—have been documented in other work with a more global focus [121], but it is important to recognize and highlight factors which may be more relevant specifically for sub-Saharan Africa. Of course, sub-Saharan itself is not a monolith and contains 46 distinct countries with a growing number of cities within these countries—across a multitude of climatic and ecological zones and each with unique characteristics and cultures. These countries are also not urbanizing at a constant or homogenous rate. Compared to the locations where studies have been conducted on the risk of urban infectious disease, Angola, Burundi, Ethiopia, Mali, and Rwanda are all rapidly urbanizing [2,122], yet relatively few studies were identified examining infectious disease risk factors among their urban populations. Geographically large countries, such as Angola, Ethiopia, and Mali may be particularly important to consider, given the concurrent challenges associated with continuing to expand primary care and basic health services to rural areas to meet universal health coverage targets, while also needing to address service provision to rapidly growing urban populations. 

Overall, the diseases covered in the identified studies largely match the primary causes of infectious premature mortality for most countries in sub-Saharan Africa, with numerous studies focused on malaria, HIV/AIDS, and diarrheal diseases. However, only five studies looked at urban tuberculosis, despite the disease being a major cause of premature morbidity and mortality in sub-Saharan Africa. The literature is also sparse for other respiratory diseases like influenza that can hold significant consequences for urban settings. Measles—as well as other vaccine-preventable diseases—continue to pose a significant threat in many sub-Saharan African countries. The rise of vaccine hesitancy [123] and increasing outbreaks in urban areas pose new challenges for interrupting transmission and ensuring sufficiently high levels of vaccination coverage [124,125]. Moreover, given the importance of tuberculosis in migrant and peri-urban communities in southern Africa [126], it may warrant additional consideration as an urban disease. 

We also identified very few articles on neglected tropical diseases such as schistosomiasis and soil-transmitted helminths, despite a recognized high prevalence of some of these parasites in urban settings and evidence that urban transmission may be increasing [127,128]. Traditional control approaches for neglected tropical diseases tend to be based on public sector-led mass drug administration, for example through school-based deworming; however, such strategies may not provide sufficient coverage or be feasible in densely populated urban settings, requiring alternative implementation strategies. 

Urban planning may be an under-utilized yet important tool, even in resource-limited settings. There are many examples where poor or absent urban planning has increased the risk of disease and work has demonstrated that the magnitude of neighborhood effects can be comparable to that of individual-level factors [107]. For example, in Lusaka, low-quality drainage is a risk factor for cholera and is associated with lower-income neighborhoods, themselves a tragic legacy of colonial-era urban planning [111]. Indeed, urban planning for public health in the 19^th^ century was largely focused on, and driven by, reducing the threat of infectious diseases to the extent that transmission patterns were understood. Modern approaches should build upon these historical efforts while basing action on improved evidence [129,130]. Such approaches, especially if combined with smaller-scale initiatives to reduce infectious disease risk at the household-level (i.e., through improved building practices), may have a large impact on reducing disease incidence and be cost-effective in the long run, when compared to the financial burden of treatment [130,131,132]. Moreover, as our analysis shows (Table 2), different diseases can share common risk factors, allowing for additive benefits across multiple diseases and disease groups if those risk factors can be holistically addressed. Given the continued dual-burden of disease faced by many African countries, and especially in heterogeneous urban environments, efforts to align urban planning with infectious disease prevention and overall health promotion should be encouraged, as well as corresponding implementation research initiatives to examine cost-benefit relationships and evidence of impact.

As seen in more global research on the links between urbanization and infectious disease, studies conducted in sub-Saharan Africa also identified high population density—a hallmark of urban life—as a critical risk factor for infectious disease. While widely acknowledged as an important variable, few studies directly examined heterogeneities in population density in their studies. The link between population density, economic opportunity, and disease may be an additional important avenue for future research, particularly given that urbanization in sub-Saharan Africa is less associated with development than in other rapidly urbanizing settings, such as in Asia [133]. Overall, there was a paucity of studies looking generally at how risk factors may vary within a city, as well as how risk factors may vary over time. Given the increasing interest in urbanization as a driver of changing epidemiological patterns, it was unexpected to find so few longitudinal studies with an urban infectious disease focus. Moreover, questions raised by Hassell and colleagues [8] related to the process of urbanization and its impact on infectious diseases have largely not been addressed, especially with respect to the importance of taking a community or systems biology approach, and incorporating One Health. Addressing these concerns and translating the significance of research findings, as well as best practices, in an intelligible fashion to urban leaders will be crucial for reducing the risk of infectious diseases in urban settings.

Beyond the pace of new studies being published outside our search timeframe, our review was subject to a number of limitations. We noted that using “urban” as a search term does not always capture studies which focus exclusively on slums or informal settlements. While we added some studies focusing on these settlements through manual supplementary searches, it may highlight an important observation of research in the field relating to urban public health research, whereby studies either treat urban areas as homogenous units, or focuses on specific sub-sections within the city. Few studies seem to explicitly consider cities as heterogeneous wholes. We also did not include risk factors relating to demographics. The link between certain diseases (e.g., HIV or malaria) and characteristics like sex or age are widely recognized, but fell beyond the scope of this review. Our categorization of diseases into groups, to facilitate a broad search, may have limited retrieval of disease-specific studies outside these groupings; in a similar vein, assignment of risk factors to these groupings may oversimplify complex dynamics between risk factors and specific diseases within those groupings. Finally, our review was limited to sub-Saharan Africa and infectious diseases. It is certain that many of the risk factors identified in global research, or that focusing on other regions, would also be applicable to sub-Saharan Africa but were not included in our review.

## 5. Conclusions

Urbanization is occurring at a rapid, but heterogeneous, pace across most countries in sub-Saharan Africa. A growing body of literature is available to better understand the human and physical geography associated with the risk of infectious diseases in urban settings. However, in the past 15 years, these have not addressed all of the countries in sub-Saharan Africa and omitted some of those that are urbanizing the quickest. While most research focuses on the major causes of morbidity, respiratory, vaccine-preventable diseases, and neglected tropical diseases may be at risk of being overlooked. Finally, future research efforts should prioritize better understanding of how urbanization is changing the epidemiology of infectious diseases, and thus impacting both rural and urban populations, as well as exploring how interventions can be adapted for implementation in the urban settings of sub-Saharan Africa.

## Figures and Tables

**Table 1:** Summary of number of studies and their locations per disease group.

Topic	Location(s) ^a^	No. Citations
Enteric disease	Chad, Ghana, Kenya, Nigeria, Senegal, Zambia, Zimbabwe	13
HIV	Benin, Botswana, Burkina Faso, Cameroon, Côte d’Ivoire, DRC, Eswatini, Ethiopia, Ghana, Guinea, Kenya, Lesotho, Liberia, Malawi, Mali, Mozambique, Namibia, Rwanda, Senegal, Sierra Leone, South Africa, South Africa, Tanzania, Zambia, Zimbabwe	34
Malaria	Angola, Benin, Burkina Faso, Cameroon, Côte d’Ivoire, DRC, Eswatini, Ethiopia, Ghana, Kenya, Madagascar, Malawi, Nigeria, Senegal, Tanzania, Uganda, Zimbabwe	38
Respiratory	Burkina Faso, Ethiopia, Guinea-Bissau, Kenya, Malawi, Niger, Nigeria, Senegal, Uganda	12
Viral hemorrhagic fever	Angola, Burkina Faso, DRC, Ethiopia, Guinea, Liberia, Nigeria, Sierra Leone	5
Other diseases ^b^	Benin, Burkina Faso, Côte d’Ivoire, Ethiopia, Kenya, Nigeria, Tanzania, Zambia, Zimbabwe	16

^a^ Some studies were conducted in multiple countries; ^b^ Other diseases included Buruli ulcer, chlamydia, gonorrhea, helminth infections, hepatitis B, herpes, Leptospirosis, Lymphatic filariasis, Schistosomiasis, and *Trichomonas vaginalis*.

**Table 2:** Summary of identified geographic and behavior risk factors per disease group. Grey fill designates where a risk factor was identified in one or more papers related to the indicated disease or disease group.

Risk Factor Category	Risk Factor	Disease or Disease Group					
		Enteric Diseases	HIV	Malaria	Respiratory Diseases	Viral Hemorrhagic Fever	Other Diseases
Geographic	Population density						
	Built environment						
	Municipal services						
	Natural environment						
Behavioral	Hygiene and sanitation						
	Education and employment						
	Sexual behaviors						
	Human movement						
	Socioeconomic standing						

## Supplementary Materials

The following are available online at https://www.mdpi.com/2414-6366/4/4/123/s1. Table S1: Search syntax. Table S2: Full details on the papers identified during the literature review, selection of possibly eligible works, full texts selected for review, and included full texts, describing the urban risk factors for infectious disease (see sequential tabs).
