# Supplementary material for: Risk Factors for Infectious Diseases in Urban Environments of Sub-Saharan Africa: A Systematic Review and Critical Appraisal of Evidence
Source: Trop Med Infect Dis. 2019 Sep 29;4(4):123. doi: 10.3390/tropicalmed4040123 (PMC6958454; doi:10.3390/tropicalmed4040123)
Supplement: Supplementary file 1 [file tropicalmed-04-00123-s001.zip › Boyce et al_supplementary materials/Table S1.docx]

**Project:** Risk factors for infectious diseases in urban environments in sub-Saharan Africa

**Purpose:** Search Syntax

**Date:** July 2019

| **Database** | **Search syntax** |
| --- | --- |
| **Literature Search Restricted From Years:**  2004–Present  **PubMed Results:**  1871 Records  **Web of Science Results:**  1739 Records | ("City" OR "Urban" OR "Urbanization")  **AND**  ("Communicable disease" OR "Infectious disease" OR “Enteric disease” OR “HIV” OR “Human Immunodeficiency Virus” OR “AIDS” OR “Acquired Immune Deficiency Syndrome Virus” OR “Acquired Immunodeficiency Syndrome Virus” OR “Malaria” OR “Plasmodium Infection” OR “Respiratory Tract Infection” OR “Respiratory Tract Infection” OR “Viral Hemorrhagic Fever”) **AND**  ("Risk" OR "Disease risk")  **AND**  ("Angola" OR "Benin" OR "Botswana" OR "Burkina Faso" OR "Burundi" OR "Cabo Verde" OR "Cameroon" OR "Central African Republic" OR "Chad" OR "Comoros" OR "Congo" OR "Democratic Republic of the Congo" OR "Republic of the Congo" OR “Congo” OR "Cote d'Ivoire" OR "Ivory Coast" OR "Equatorial Guinea" OR "Eritrea" OR "Eswatini" OR "Swaziland" OR "Ethiopia" OR "Gabon" OR "Gambia" OR "Ghana" OR "Guinea" OR "Guinea-Bissau" OR "Kenya" OR "Lesotho" OR "Liberia" OR "Madagascar" OR "Malawi" OR "Mali" OR "Mauritania" OR "Mauritius" OR "Mozambique" OR "Namibia" OR "Niger" OR "Nigeria" OR "Rwanda" OR "Sao Tome and Principe" OR "Senegal" OR "Seychelles" OR "Sierra Leone" OR "South Africa" OR "South Sudan" OR "Tanzania" OR "Togo" OR "Uganda" OR "Zambia" OR "Zimbabwe" OR "Zanzibar") |
